# Quantifying Tremor in Essential Tremor Using Inertial Sensors—Validation of an Algorithm

**DOI:** 10.1109/JTEHM.2020.3032924

**Published:** 2020-10-21

**Authors:** Patrick Mcgurrin, James Mcnames, Tianxia Wu, Mark Hallett, Dietrich Haubenberger

**Affiliations:** 1 National Institute for Neurological Disorders and Stroke, National Institutes of HealthBethesdaMD20892USA; 2 Department of Electrical and Computer EngineeringPortland State University 6685 PortlandOR97201USA; 3 Office of the Clinical DirectorNational Institute for Neurological Disorders and Stroke, National Institutes of HealthBethesdaMD20892USA

**Keywords:** Essential tremor, inertial sensor, movement disorders, TETRAS

## Abstract

*Background* Assessment of essential tremor is often done by a trained clinician who observes the limbs during different postures and actions and subsequently rates the tremor. While this method has been shown to be reliable, the inter- and intra-rater reliability and need for training can make the use of this method for symptom progression difficult. Many limitations of clinical rating scales can potentially be overcome by using inertial sensors, but to date many algorithms designed to quantify tremor have key limitations. *Methods* We propose a novel algorithm to characterize tremor using inertial sensors. It uses a two-stage approach that 1) estimates the tremor frequency of a subject and only quantifies tremor near that range; 2) estimates the tremor amplitude as the portion of signal power above baseline activity during recording, allowing tremor estimation even in the presence of other activity; and 3) estimates tremor amplitude in physical units of translation (cm) and rotation (°), consistent with current tremor rating scales. We validated the algorithm technically using a robotic arm and clinically by comparing algorithm output with data reported by a trained clinician administering a tremor rating scale to a cohort of essential tremor patients. *Results* Technical validation demonstrated rotational amplitude accuracy better than ±0.2 degrees and position amplitude accuracy better than ±0.1 cm. Clinical validation revealed that both rotation and position components were significantly correlated with tremor rating scale scores. *Conclusion* We demonstrate that our algorithm can quantify tremor accurately even in the presence of other activities, perhaps providing a step forward for at-home monitoring.

## I. Introduction

Essential tremor (ET) is one of the most common movement disorders. It is characterized by 4–12 Hz rhythmic shaking of the hands and arms. Tremor in ET patients is brief, occurs primarily during action, and changes continuously. The tremor makes it challenging for people to perform activities of daily living such as drinking from a glass and writing [1]–[3].

The Essential Tremor Rating and Assessment Scale (TETRAS) is the most widely accepted standard for clinical assessment and quantification of ET severity. It includes a clinician-rated performance scale assessing tremor severity, quantified by amplitude or impact on a prescribed task such as handwriting or spiral drawing [4], [5]. During the administration of TETRAS performance scale, a clinician observes the participant’s limbs during different postures and activities. The clinician scores the limb displacement due to tremor in units of centimeters using a scale from 0 to 4 (with higher number referring to a higher amplitude tremor).

While TETRAS has been shown to be a valid and reliable clinical tool for characterizing postural and kinetic tremor, like all clinician-reported outcomes, it has significant inter-rater and intra-rater variability due to differences in interpretation and perception of the tremor [5], [6]. This diminishes the value of TETRAS in both clinical practice and clinical trials for tracking the progression of symptoms over time and determining the effectiveness of interventions.

Rating scales like TETRAS must be administered by clinicians who have been properly certified to administer and score them. Clinical or outpatient centers must be staffed with trained and qualified personnel, or alternatively have dedicated equipment to video record and/or use internet-based means to connect to an expert. The cost for using rating scales like TETRAS can also be significant when fees for training, certification, licensing, and administration are accounted for, particularly in the context of clinical trials.

Many of the limitations of clinical rating scales can be overcome with wearable devices that include inertial sensors. With a proper algorithm, these devices can assess tremor during prescribed activities and postures like those used for clinical rating scales as well as during normal daily activities. Modern inertial sensors are sufficiently accurate that the intra-device and inter-device variability is an insignificant source of variability. They are typically high precision (14 bits or better), sample at rates well above that required to characterize tremor (≈25 Hz), and have a wide dynamic range: typically ±15 g or more for accelerometers and ±1500

There have been many algorithms proposed to quantify tremor from inertial sensors. Recent reviews can be found in [7]–[9]. Additionally, some groups have used machine learning algorithms to quantify tremor [10], [11], and many groups have evaluated the potential of commercial off-the-shelf smart watches and smart phones [12]–[15]. However, much of the algorithm development has focused on rest tremor, which is common to Parkinson’s disease (PD). These algorithms are typically compared to the Unified Parkinson’s Disease Rating Scale (UPDRS). For example, Van Someren successfully discriminated tremor from other movements. Reference [16], applying this approach via a custom actigraph with a single-axis accelerometer to achieve a between-subject correlation of 0.96 to item 20 of the UPDRS [17]. In later work, they embedded a tremor detection algorithm in an activity monitor and attained a correlation of 0.93 with the average UPDRS tremor score [18]. Hoff *et al.* used a triaxial accelerometer mounted to the most affected wrist to study tremor, attaining a sensitivity ≥ 0.82 and specificity ≥ 0.93, as compared to video review [19]. Similarly, Salarian *et al.* used triaxial gyroscopes attached to the wrists to quantify tremor [20], [21], attaining an overall sensitivity of 99.5% based on video review and a specificity of 94.2% using age-matched controls.

Assessing tremor in people with ET is more challenging relative to PD tremor because they predominantly experience action tremor that occurs while some other activity is being performed. Activities that elicit action tremor often contain abrupt movements that occur over a broad frequency range that overlap with the frequency bands that include tremor. Algorithms that attribute all of the signal power within the tremor frequency bands to tremor over-estimate the tremor amplitude. These algorithms can generate false positives when the activities performed are rhythmic at a frequency that is within the detection frequency range of the algorithm.

In addition, action tremor typical of ET is intermittent, as it is dependent on underlying background-activity. Although ET is characterized by an average frequency in the range of 4–12 Hz across people, the range of the tremor frequency occurs over a much narrower range, ≈ ±1 Hz, for each individual subject, and may vary across tasks within subjects [22], [23]. The tremor may begin and end abruptly depending on the activity of the subject. When it is present for a sustained period, the tremor frequency changes continuously.

Previous algorithm designs have largely ignored these properties of tremor that are specific to action tremor common to ET [12]. While some recent work has shown a correlation between a portable smartwatch tremor recording device and both the Fahn-Tolosa-Marin Tremor Rating Scale [12], [13] and the Washington Heights-Inwood Genetic Study of Essential Tremor Tremor Rating Scale [24], these algorithms did not account for these properties of the action tremor common to ET.

Some algorithms have focused dominantly on the ability to detect tremor without regard to the amplitude necessary for tremor to be considered clinically significant [19], [20]. Others have used units for tremor amplitude that are not meaningful to clinicians, patients, clinical trialists, and scientists. These units include g-forces (m/s^2^) recorded from an accelerometer [17], the power of the tremor frequency [10], [18], and both the root mean squared error and standard deviation of angular velocity [10]. In contrast, most clinical rating scales, including TETRAS, are based on amplitude measures that are in units of centimeters. However, it is difficult to estimate tremor in physical units from inertial sensors because the units of the sensors are far removed from physical and rotational displacement, which are more meaningful. Gyroscopes measure rotational velocity. Accelerometers measure specific force, which includes the effects of both the translational acceleration and gravity combined [25]. This is especially problematic for algorithms based on accelerometers alone in which rotation and translational movements can be indistinguishable.

We propose a new algorithm based on wearable devices with inertial sensors that overcomes these limitations of previous algorithms. The proposed algorithm uses a two-stage approach to cover the entire frequency range of tremor expected for a disease population, while also reducing false positives by using a narrower detection range for each individual during the second stage. We previously introduced this two-stage approach for a simpler algorithm for passive monitoring [26].

The proposed algorithm estimates the tremor amplitude in physical units (degrees and centimeters) that are easily understood and interpreted by patients, participants, clinicians, and scientists. It was designed to accurately detect both action and rest tremors, and can indicate which type of tremor is detected. We provide a complete description of the proposed algorithm that includes all of the processing steps and parameter values.

We rigorously validated the algorithm to assess its construct validity. We used an industrial robotic arm for technical validation. This served as a gold standard reference in which the frequency and amplitude of the tremor was precisely controlled and provides evidence of criterion validity. We also performed a clinical validation by comparing the algorithm to TETRAS, as has been done in previous work. This provides evidence of concurrent validity.

## II. Methods and Procedures

### A. Algorithm Design

#### 1) Stage 1: Spectral Peak Detection

The first stage of the algorithm uses a sliding window approach to find candidate tremor peaks in the power spectral densities of both the translational acceleration and the rotational velocity. The following steps are applied to all of the available recordings from sensors at all available locations on the body. During the first stage all spectral peaks are considered that are within the range of expected tremor frequencies for the subject population. For example, in ET patients an appropriate frequency range is 4–12 Hz [2].

**Fig. 1.**
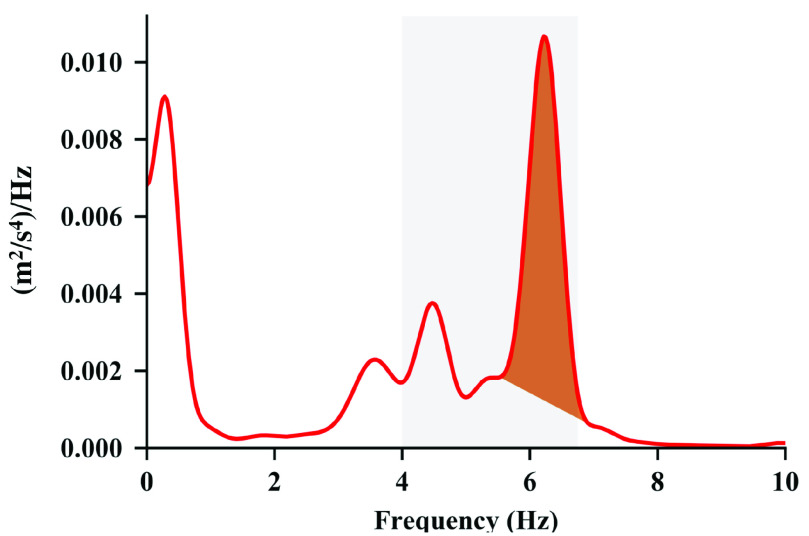
Example of a spectral peak in the power spectral density (PSD) that is attributed to tremor. The gray shaded region shows the expected bounds of the tremor frequency from one subject. The power attributed to tremor is limited to the shaded region that is above the straight-line estimate of the baseline activity.

**Fig. 2.**
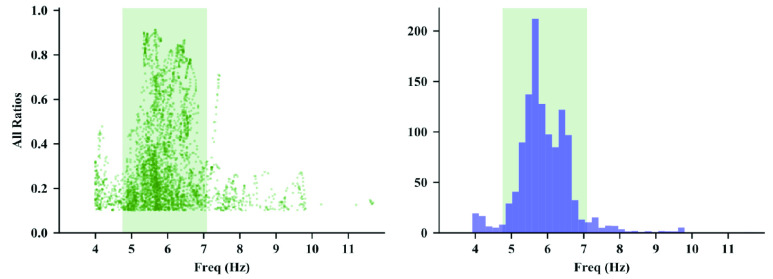
Left plot shows the power fractions for all of the recordings at all sensor locations for one of the subjects. The right plot shows a weighted histogram of the power fractions. The green shaded region in each plot shows the estimated range of tremor frequencies expected for this subject.

Step 1.1. Determine the orientation of the inertial sensor in the Earth frame. We use the right-handed reference frame of North-West-Up for $x$, $y$, and $z$. Most companies that manufacture and produce inertial sensors have a proprietary algorithm for estimating this orientation. The algorithm is often called an attitude and heading reference system (AHRS). The principles underlying this algorithm are well known [27]–[29].
Step 1.2. Use the orientation to transform the accelerometer signals into the Earth reference frame and subtract gravity, similar to the approach in [27]–[30]. This is a more accurate and general way to separate the gravitational component from translational acceleration than earlier approaches based on constrained models of movement [25].
Step 1.3. Calculate a spectrogram of the acceleration representing the PSD with a sliding Blackman window. We applied a Blackman-Tukey nonparametric PSD to each windowed segment. The PSD is computed separately for each channel and then the individual PSDs are summed together to calculate the total PSD for acceleration. The total PSD calculated in this way is invariant to the orientation of the sensor relative to the Earth reference frame so it does not matter how the sensor is oriented on the body.
Step 1.4. Calculate the spectrogram of the rotational velocity as measured from the gyroscopes using the same approach.
Step 1.5. For each windowed segment and for each of the two types of spectrograms (translational acceleration and rotational velocity), detect all of the spectral peaks. The beginning and end of each peak is expanded until one of three stopping criteria are met:
  1) The maximum allowed peak width is reached
  2) The beginning and end of the peak reaches their local minima
  3) The PSD drops below a straight line connected the beginning and end of the peak as shown in Figure 1
Step 1.6. Calculate the power fraction of each spectral peak
  \begin{equation*} \rho = \frac {\int _{f_{\mathrm {b}}}^{f_{\mathrm {e}}} p(f) - b(f) \thinspace \mathrm {d}f}{\max \left ({p_{\min },\int _{f>f_{\mathrm {T}}} p(f) \thinspace \mathrm {d}f }\right)}\tag{1}\end{equation*}
  where $\rho $ is the proportion of power attributed to the spectral peak, $p(f)$ is the PSD of the time slice, $f_{\mathrm {b}}$ is the start of the spectral peak, $f_{\mathrm {e}}$ is the end of the spectral peak, $b(f)$ is the estimated straight-line baseline below the spectral peak, $p_{\min }$ is the smallest power that would be considered as significant movement, $f_{\mathrm {T}}$ is a frequency threshold that represents the smallest frequency at which the tremor may contain some power. This is illustrated in Figure 2.
  The subtraction of the estimated baseline $b(f)$ in the PSD removes the effect of other activity that may have spectral power over the same frequency range as the tremor. This is more accurate than most methods that assume all of the power associated with a peak is due to tremor, as well as methods that assume all of the spectral power is contained in discrete peaks [31].
  The power fraction estimates the power attributed to the peak relative to either the total power over the frequency range of interest ($f>f_{\mathrm {T}}$) or the minimum power that would be considered as significant, whichever is greater. This approach ensures candidate spectral peaks are not detected with insignificant power and provides a figure of merit that prioritizes the peaks that represent the largest proportion of signal power. This is effective in both recordings with action tremor in which other activity is present and during recordings of rest and postural tremor in which the tremor is the dominant activity. This prevents over-estimation of the tremor amplitude that may arise due to signal power attributed to either action or posture that may generate rhythmic activity at a frequency that is within the detection frequency range of the algorithm.
Step 1.7. For each windowed segment find the spectral peak with the largest power fraction that is within the bounds of the expected tremor frequency, $f_{\mathrm {l}} \leq f \leq f_{\mathrm {u}}$. This spectral peak may occur in either the PSD for the acceleration or the rotational velocity. This represents the best estimate of the tremor frequency for this segment.
Step 1.8. Determine whether other non-tremor activity is present. Calculate the total spectral power of the position from the acceleration spectral power by performing integration in the frequency domain:
  \begin{equation*} p_{p}(f) = \frac {p_{a}(f)}{\left ({2\pi f}\right)^{4}}\tag{2}\end{equation*}
  where $p_{a}(f)$ is the spectral power of the translational acceleration in the Earth frame and $p_{p}(f)$ is the spectral power of the translational position in the Earth frame. The operation of integration in the time domain is equivalent to dividing the Fourier transform by $2\pi f$. The term in the denominator is taken to the fourth power because the integration is performed twice to estimate position from acceleration and because this equation relates the power spectral densities rather than the signal transforms. The total power associated with action is calculated as
  \begin{equation*} p_{a,\text {rms}} = \sqrt {\int _{f_{a,\min }}^{f_{a,\max }} p_{p}(f) \thinspace \mathrm {d}f}\tag{3}\end{equation*}
  where $f_{a,\min }$ and $f_{a,\max }$ represents the frequency range in which action is typically dominant. If the root mean square amplitude of the signal exceeds a threshold, declare action as detected.

#### 2) Stage 2: Tremor Frequency Bound Estimation:

The second stage of the algorithm uses all of the candidate spectral peaks and their power fractions to estimate the expected range of tremor frequencies for this specific subject. It’s important to note that while the algorithm estimates the frequency range for each subject individually, the key algorithm parameters were applied to all subjects (see Table 1).

**TABLE 1:** Summary of the Key Algorithm Parameters

Parameter Description	Value
Spectrogram Sliding Window Duration	4.0 s
Spectrogram Update Interval	0.1 s
Autocorrelation Window Duration	3.5 s
Spectral Peak Minimum Width	0.8 Hz
Spectral Peak Maximum Width	1.8 Hz
Population Tremor Frequency Lower Bound ($f_{\mathrm {l}}$)	4.0 Hz
Population Tremor Frequency Upper Bound ($f_{\mathrm {u}}$)	12.0 Hz
Second Stage Tremor Frequency Range about Median	±1.5 Hz
Minimum Power Faction ($\rho _{\min }$)	0.10
Power Faction Detection Threshold ($\rho _{\mathrm {d}}$)	0.20
Interquartile Range Excess ($\delta _{f}$).	0.75 Hz
Smallest Action Frequency ($f_{\min }$)	1.0 Hz
Largest Action Frequency ($f_{\max }$)	3.5 Hz
Action Detection Threshold	0.0025 m/sA^2^

Step 2.1. Sort all of the power fractions that are greater than some minimum $\rho _{\min }$ in order of increasing frequency such that $\rho _{i}$ is the power fraction for the spectral peak with a peak frequency of $f_{i}$
Step 2.2. Calculate the normalized cumulative sum of power fractions,
  \begin{equation*} w_{j} = \frac {\sum _{i=0}^{j} \rho _{i}}{\sum _{i=0}^{n_\rho } \rho _{i}}\tag{4}\end{equation*}
  where $w_{j}$ is the normalized cumulative sum through the $j$th power fraction, $\rho _{i}$ is the power fraction, and $n_\rho $ is the number of spectral peaks that were detected.
Step 2.3. Find the 25th and 75th percentiles of the cumulative sum,
  \begin{align*} j_{25}=&\mathop {\mathrm {arg\,max}} _{j} w_{j} < 0.25 \tag{5}\\ j_{75}=&\mathop {\mathrm {arg\,min}} _{j} w_{j}>0.75\tag{6}\end{align*}
  Collectively these represent the interquartile range of the distribution of spectral peak frequencies weighted by the power fractions.
Step 2.4 Estimate the tremor frequency range for this subject,
  \begin{align*} f_{\mathrm {l}} = f_{j_{25}} - \delta _{f} \tag{7}\\ f_{\mathrm {u}} = f_{j_{75}} + \delta _{f}\tag{8}\end{align*}
  where $f_{\mathrm {l}}$ is the lower bound of the expected tremor frequency range for this subject, $f_{\mathrm {u}}$ is the upper bound of the expected frequency range for this subject, and $\delta _{f}$ is the amount outside of the interquartile range that tremor might be expected. The interquartile range with an offset was used to ensure the estimated frequency range for the subject was insensitive to detection outliers.

#### 3) Stage 3: Tremor Detection and Characterization

This final stage of the algorithm makes a second pass through all of the recordings and sensor locations for the subject to find the spectral peaks that are within the tremor frequency bounds identified in Stage 2. This ensures that the frequency range is not arbitrarily selected based on the broad range of any one tremor type, for example for that of Parkinson’s Disease or essential tremor.

Step 3.1. With the new frequency bounds identified in Stage 2, repeat Step 1.8 for all recordings, segments, and sensors to identify the most promising spectral peaks.
Step 3.2. If the power fraction is larger than a tremor detection threshold $\rho _{\mathrm {d}}$, then declare tremor as detected and estimate the rotation amplitude and position amplitude from the power spectral densities. The amplitude estimate is based on modeling power in the spectral peak as a sinusoid. In this case, power contained in the spectral peak is expected to be $\frac {1}{2} a^{2}$ where $a$ is the amplitude of the sinusoid. This means the amplitude of the underlying sinusoid can be estimated as
  \begin{equation*} a \approx \sqrt {2.0 \int _{f_{\mathrm {b}}}^{f_{\mathrm {e}}} p(f) - b(f) \mathrm {d}f}\tag{9}\end{equation*}

To estimate the translational amplitude in units of position rather than acceleration, we can approximate double integration of the acceleration in the frequency domain so that the translational amplitude is

\begin{equation*} a_{p} = \frac {\sqrt {2.0 \int _{f_{\mathrm {b}}}^{f_{\mathrm {e}}} p(f) - b(f) \mathrm {d}f}}{(2\pi f)^{2}}\tag{10}\end{equation*}

Similarly, the rotational amplitude is estimated as an approximate single integral of the rotational velocity in the frequency domain

\begin{equation*} a_{r} = \frac {\sqrt {2.0 \int _{f_{\mathrm {b}}}^{f_{\mathrm {e}}} p(f) - b(f) \mathrm {d}f}}{(2\pi f)}\tag{11}\end{equation*}

**Fig. 3.**
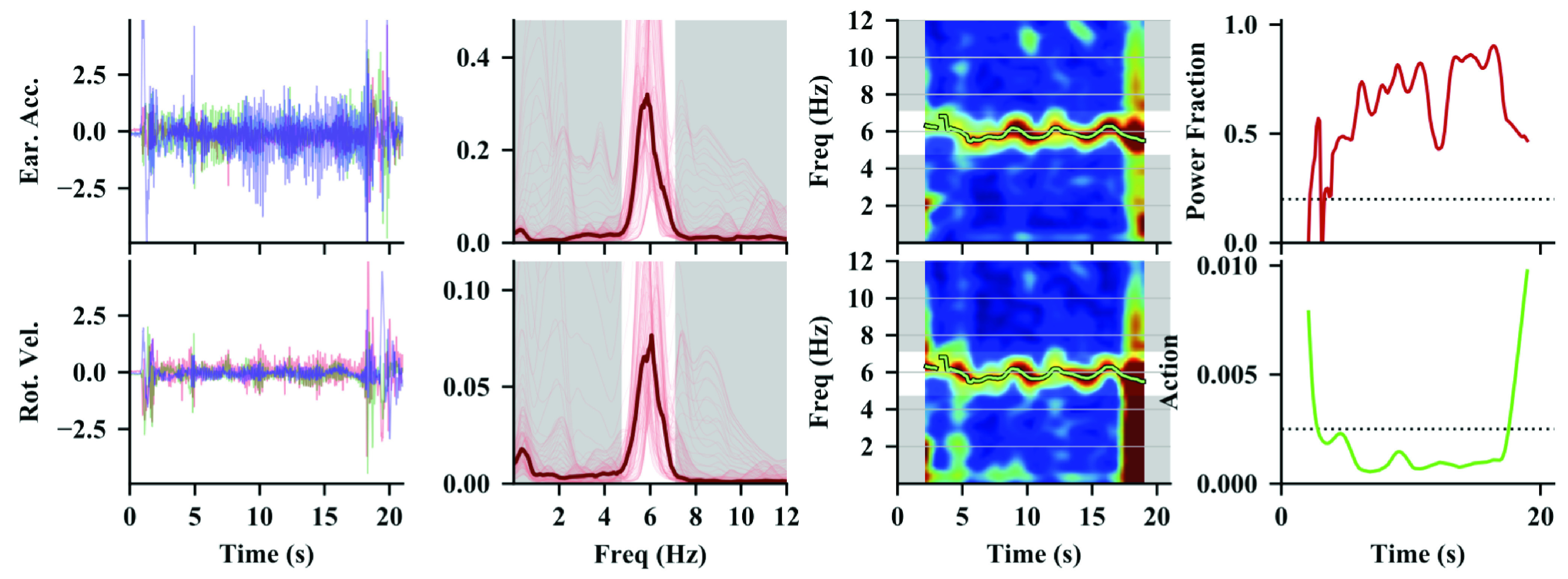
The top row of plots shows the algorithm as applied to the acceleration in the Earth frame and the bottom row shows the algorithm as applied to the rotational velocity. The left column of plots show the time-domain signals. The second column of plots shows the median power spectral density (PSD) as the thick, dark red line. The individual PSDs from each windowed segment are shown by the thin red lines. The third column of plots shows the spectrogram amplitudes. The green line shows the estimated tremor frequency. The plot in the top right corner shows the power fraction of the largest spectral peak within the frequency bounds for this subject (red trace). The plot in the bottom right corner shows the power associated with action (green trace). The thresholds for detection are shown by the horizontal dashed lines.

These amplitudes can then be expressed in physical units typical of tremor, such as centimeters and degrees. Figure 3 shows an example of the algorithm as applied to a single recording at a single location.

The key design decisions and parameter values that we used for the results reported in this paper are listed in Table 1. These values were chosen based on the known properties of tremor in the subject population, previous studies, and empirically from the visualizations of the sensor data alone. The results in Section III were evaluated independently after the algorithm design and parameter values were finalized.

### B. Technical Validation

**Fig. 4.**
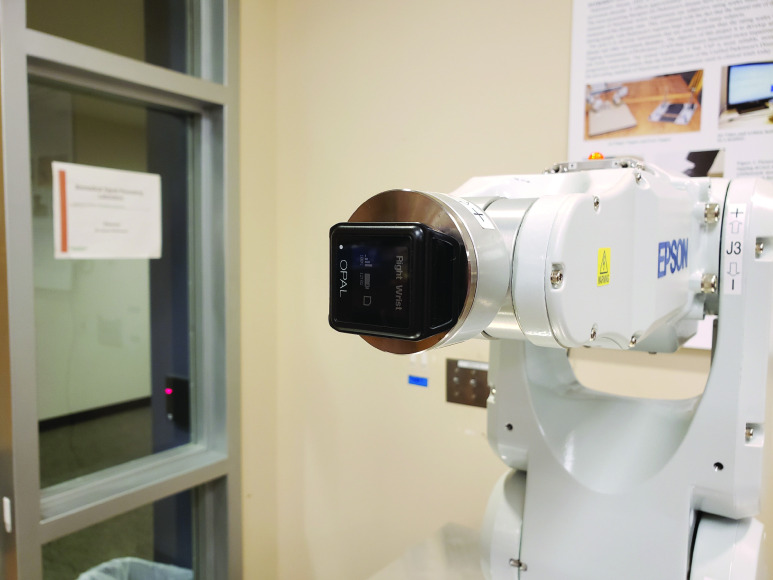
Epson C3 robotic arm used for algorithm verification. The inertial sensor is attached to the end effector using a custom 3D-printed jig.

To verify that the estimates of the rotational and translational tremor amplitudes were accurate, we evaluated the algorithm with an industrial Epson C3 robotic arm (Epson Robots, Carson, California). The arm has six degrees of freedom, as shown in Fig. 4, and is capable of movements that are rapid, precise, and repeatable. Although the arm was designed for industrial applications, it is well suited for controlled studies of movement. Optical motion capture systems are often used to measure position of reflective markers with high precision (<1 mm), but they do not provide any means of precise and repeatable control of movement. The C3 provides angular velocities of each of the six joints in the range of 450–720 °/s. It has a repeatability of ±0.02 mm and a work area of $\mathrm {\pm.48 \times \pm.48 \times \pm.48~m}$.

The robotic arm was programmed to model rotational and positional tremor over a frequency range that the arm was capable of (4.0–6.4 Hz) for several rotation and position amplitudes similar to what would be observed during tremor. This provided a precise reference for the inertial sensor and tremor algorithm. The inertial sensor was attached to the end effector with a custom jig that was designed and 3D printed for this purpose. Figure 4 shows a picture of the inertial sensor attached to the robot arm.

### C. Clinical Validation

Inertial sensors (APDM Opal, Portland, Oregon, USA; $43.7\times 39.7\times13.7$ mm (LxWxH); 22 grams) were placed bilaterally on the dorsum of each hand, shanks, and the lumbar spine. Short (15.5 s ± 6.0 s, $\mu \pm \sigma $, $n=182$) recordings were acquired with the inertial sensors for each item of the TETRAS Performance clinical rating scale while a clinician also evaluated the patient. This ensured that the inertial sensor recording and the clinical evaluation happened concurrently. TETRAS performance ratings were manually recorded at the time of each recording. Inertial sensor data was labelled and saved for offline processing. The same algorithm used for the robot arm validation step was used to process each inertial sensor recording collected when administering the TETRAS.

The sensor location was selected based on where the tremor was visually rated for each item. For kinetic, posture, dot, spiral, handwriting, and wingbeat the hand sensor was used for the corresponding side (e.g., right kinetic was compared to the tremor observed at the right hand). For standing, the lumbar sensor was used. For lower limb, the max sensor value from the right and left leg sensors was used from the postural and kinetic conditions. We isolated the comparison of tremor from one sensor to one metric of TETRAS because this matches how the TETRAS would be administered in a clinical setting.

As part of the TETRAS exam, patients were also asked to draw Archimedes spirals as an additional metric to quantify tremor. Spirals were drawn holding a pen with either hand while preventing the hand and arm from making contact with the page.

Spearman correlations were used to assess the relationship between the rotational and translational components of the inertial sensor data with the TETRAS. The first analysis compared all data together, and subsequent analyses evaluated the relationship of each individual component of the TETRAS. In addition, mean rotational and translational scores (averaged over all 13 conditions of TETRAS) were correlated with the total TETRAS score.

#### 1) Subject Information

Thirteen patients, 63± 16 years of age (9 female), gave their written informed consent, and the protocol was approved by the National Institutes of Health Combined Neurosciences Institutional Review Board. All subjects had a previous diagnosis of classic ET per the Movement Disorder Society 1998 classification [2]. The average age of onset was 27± 17 years of age, with an average disease duration of 36± 10 years (see Table 2). Five of the 13 patients were taking medication for ET, and the medication was not paused for the study. A full history and physical exam were performed for each patient on the day of the study. The patient demographics are listed in Table 2.

**TABLE 2:** Patient Demographics

ID	Gen	Age	Age of Onset	Dis. Dur.	Med	TETRAS Total Performance (Max 52)
1	M	78	43	35	No	22.0
2	F	81	50	31	No	27.0
3	F	67	20	47	Yes	23.0
4	F	45	10	35	No	20.0
5	M	67	15	52	Yes	21.0
6	M	82	45	37	Yes	17.5
7	F	65	20	45	No	23.0
8	F	50	12	38	No	26.5
9	F	60	14	46	Yes	26.5
10	F	71	40	31	No	16.0
11	F	80	62	18	Yes	19.5
12	M	70	17	53	No	19.0
13	F	24	5	19	Yes	38.0

## III. Results

### A. Technical Validation

The results of verification with prescribed movements by the robotic arm are listed in Tables 3 and 4. There was excellent overall agreement between the estimated and actual amplitudes. Specifically, there was less than 0.05 degrees difference in the estimated rotational amplitude and 0.02 cm in translational amplitude.

**TABLE 3:** Summary of the Rotational Tremor Performance

Frequency Hz	Specified °	Estimated °
4.3	0.50	0.46
5.3	0.50	0.48
6.4	0.50	0.49
7.1	0.50	0.50
8.6	0.50	0.54
4.2	1.00	0.94
5.1	1.00	0.97
6.4	1.00	1.02
4.0	2.50	2.43
4.9	2.50	2.65

**TABLE 4:** Summary of the Positional Tremor Performance

Frequency Hz	Specified cm	Estimated cm
4.0	0.50	0.55
5.1	0.50	0.56
5.8	0.50	0.57
5.3	0.25	0.28
5.9	0.25	0.28
6.4	0.25	0.29

### B. Clinical Validation

**Fig. 5.**
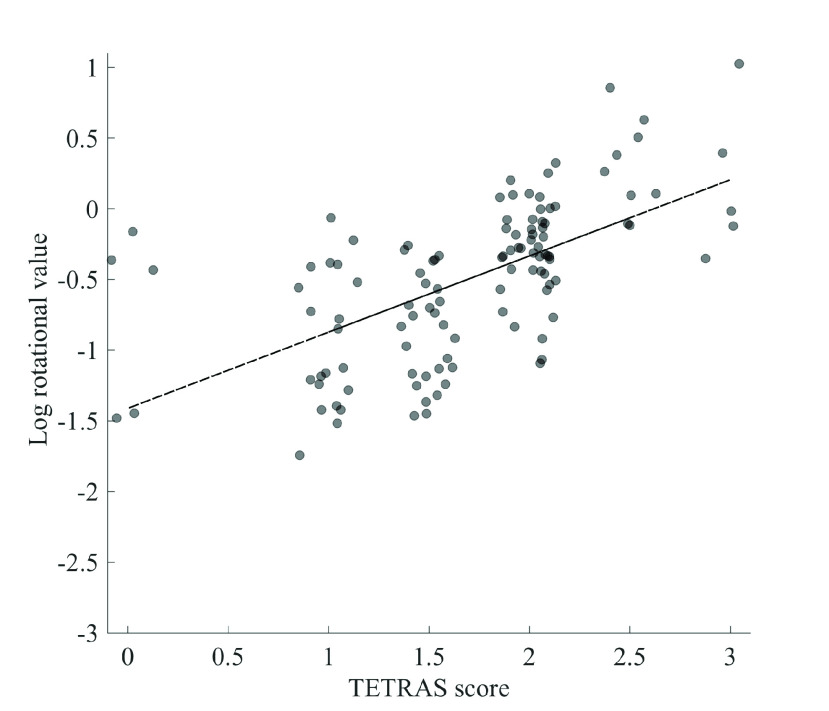
Log rotational sensor data as a function of TETRAS performance score for all TETRAS values, irrespective of condition subcategory.

**Fig. 6.**
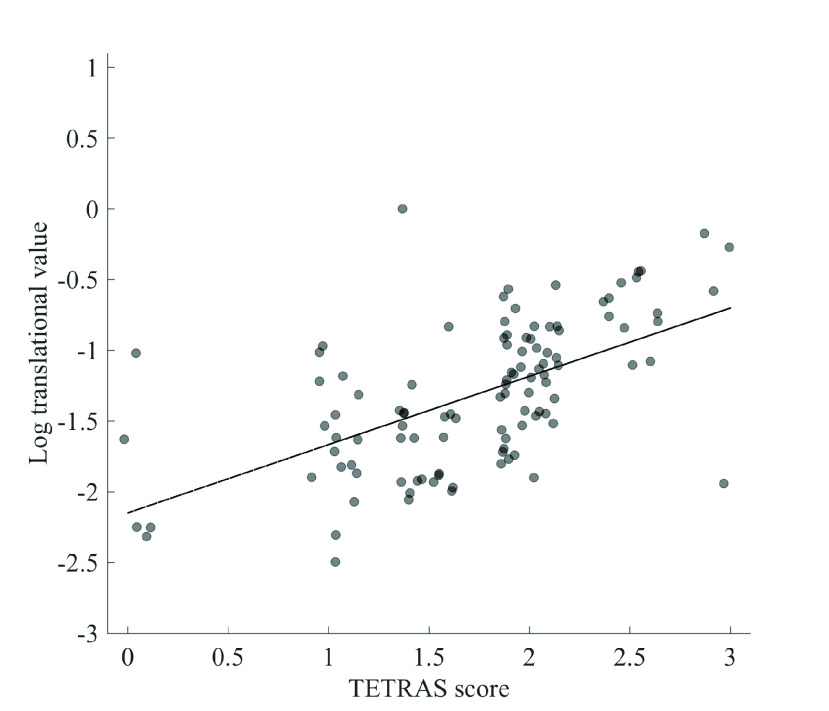
Log translational sensor data as a function of TETRAS performance score for all TETRAS values, irrespective of condition subcategory.

The average TETRAS performance score for all patients was 23±5.68 (mean±SD; see Table 5). We found a significant positive relationships between the TETRAS performance and inertial sensor metrics (see Fig. 5 and Fig. 6), including both the rotational (0.683, $p < 0.001$) and translational (0.641, $p < 0.001$) components.

**TABLE 5:** Relationship Between TETRAS Scores and Inertial Sensor Data. The Columns Include the Mean ($\mu$), Standard Deviation ($\sigma$), Standard Error of the Mean ($\sigma_{\mu}$), Spearman Correlation Coefficient ($\rho$), and $p$-Value for Statistical Significance of the Correlation Coefficient ($p$)

TETRAS	TETRAS Perf.			Position		Rotation	
Item	$\mu $	$\sigma $	$\sigma _{\mu }$	$\rho $	$p$	$\rho $	$p$
R Postural	1.46	0.56	0.16	0.772	0.043	0.342	0.302
L Postural	1.50	0.46	0.13	0.512	0.267	0.780	0.008
R Wingbeat	1.69	0.43	0.12	0.702	0.041	0.833	0.001
L Wingbeat	1.81	0.48	0.13	0.577	0.143	0.718	0.012
R Kinetic	2.04	0.32	0.09	0.139	0.762	0.949	0.167
L Kinetic	2.19	0.25	0.07	−0.282	0.571	0.866	0.667
Lower Limb	1.23	0.93	0.23	−0.026	0.937	−0.055	0.866
R Spiral	1.85	0.38	0.10	0.548	0.222	0.671	0.024
L Spiral	1.92	0.49	0.14	0.348	0.324	0.372	0.259
Handwriting	1.77	0.83	0.23	0.661	0.124	0.926	0.033
R Dot	1.88	0.46	0.13	0.715	0.020	0.489	0.127
L Dot	1.96	0.32	0.09	0.327	0.327	0.589	0.073
Standing	0.17	0.58	0.16	0.775	0.500	0.866	0.667
Total	23.0	5.68	1.58

**Fig. 7.**
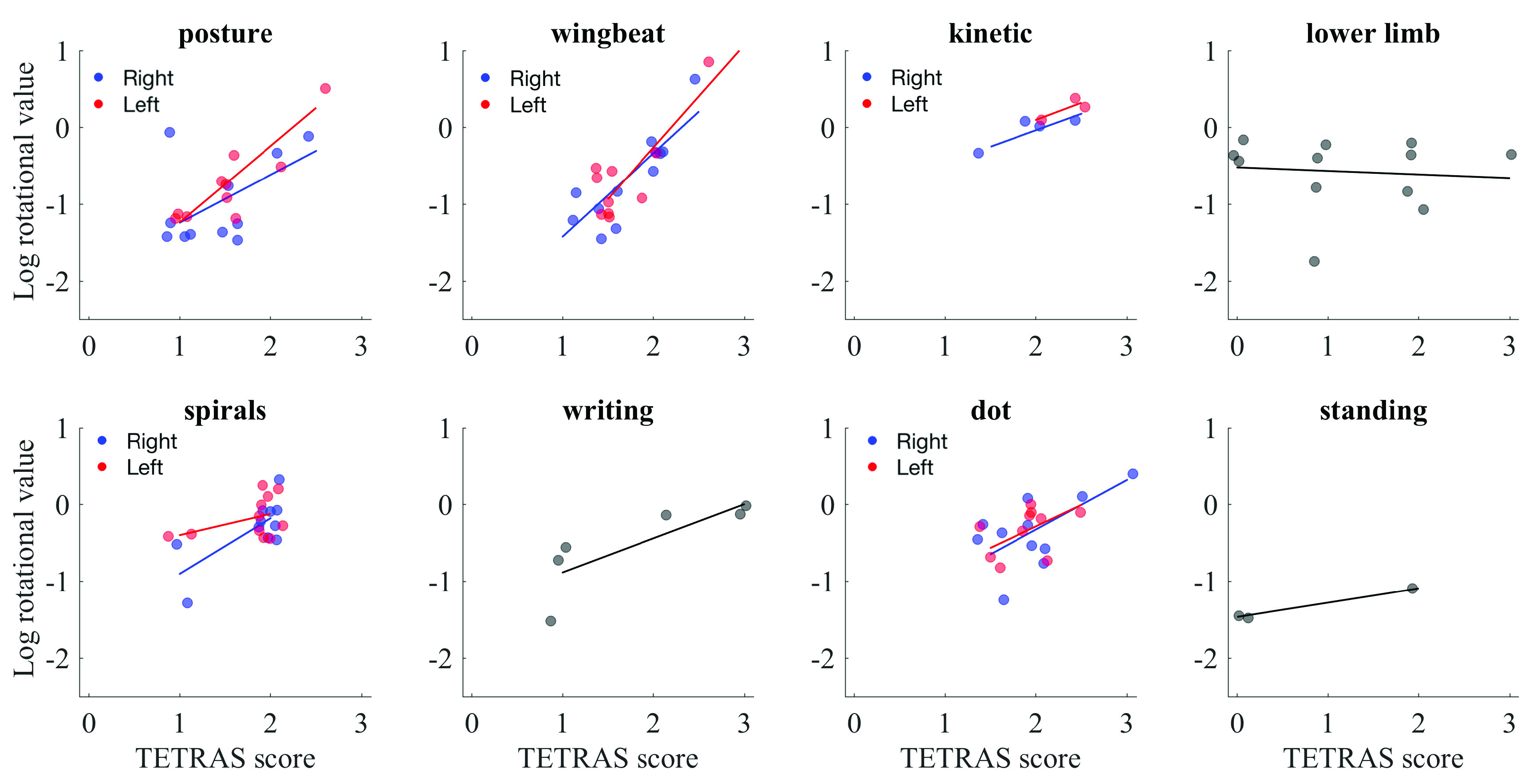
Log rotational sensor data as a function of TETRAS score for each condition. Values in which both left and right side data were collected are split into two data sets and indicated in red and blue. Gray dots represent data for which data was collected either from the dominant side or in which it was collected from max of the lower limb.

Further analysis was also performed to compare individual conditions of the TETRAS scale (see Table 5). For the rotational data, there was a significant relationship between TETRAS and inertial sensor data in the left postural (0.780, $p = 0.008 0$), right, wingbeat (0.833, $p = 0.001$), left wingbeat (0.718, $p = 0.012$), and handwriting (0.926, $p = 0.033$) items (see Fig. 7).

The sample size was moderate so there were more items with a moderate-to-strong correlation than there were that were statistically significant. For example, left hand postural, right/left wingbeat, right/left kinetic, right spirals, left dot approximation, handwriting, and standing all had correlations of 0.5 or greater.

For the translational data, a significant relationship between TETRAS and inertial sensor data in the right postural (0.772, $p = 0.043$), right, wingbeat (0.702, $p = 0.041$), and right dot approximation (0.715, $p = 0.020$) conditions. Left/right hand postural, left/right wingbeat, handwriting, right spiral, right dot approximation, and standing met criteria for having a correlation of 0.5 or greater.

**Fig. 8.**
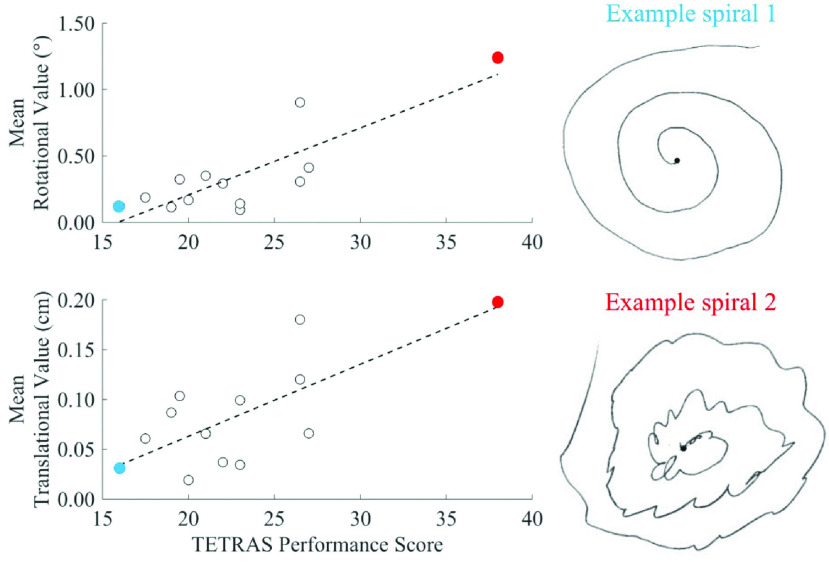
Spiral data compared with TETRAS performance and algorithm data. Mean rotational and translational data were plotted against total TETRAS performance score. Spirals were selected and plotted for the patients with the highest and lowest TETRAS score. These data points are indicated on the scatter plots using red and blue colors, respectively.

We also compared the total TETRAS Performance score with the average rotational and translational amplitudes. There was a significant relationship between the mean rotational (0.592, $p = 0.032$) and translational (0.576, $p = 0.039$) averages with total TETRAS Performance score (see Fig. 8). This figure also shows a comparison of the inertial sensor and spiral data for the most extreme subjects (TETRAS scores of 38 and 16, respectively). The patient with the highest TETRAS score also had the highest mean rotational and mean translational values when reviewing the inertial sensor data. Similarly, the patient with the lowest TETRAS score had the lowest mean rotational and mean translational values. Spirals were selected and plotted for the patients with these highest and lowest TETRAS score for visual comparison.

## IV. Discussion

Overall, the algorithm correlated well with clinical scores of tasks such as the dot approximation, spiral drawing, and handwriting tasks. This is likely because the tremor was present continuously during each of these tasks, even if the frequency and amplitude of the tremor varied slowly over the duration of the task.

In contrast, the tremor algorithm did not have a statistically significant relationship with the finger-nose-finger task. We believe that this is because the raters who apply TETRAS are instructed to score the “highest amplitude seen at any point during the exam” for each task. The finger-nose-finger task requires the subject to repeatably move their finger between the examiner’s finger and the subject’s nose. This task includes three different types of movement: voluntary movement, movement termination, and maintaining the finger at the target location. Each of these movement types may elicit a different type of tremor: simple kinetic tremor, intention tremor, and postural tremor, respectively [2]. Often the strongest observed amplitude occurs as the finger reaches approaches the target and is only includes a couple of oscillations. Human raters are arguably able to estimate the amplitude of intention tremor because they can use a mental model of how a subject without tremor would perform the task in a manner that would not include any overshoot. It is also possible that dysmetria confounded the raters resulting in inflated scores [32].

Although the new algorithm is designed to estimate tremor in the presence of other types of movement, it is not well suited for tasks in which the tremor of interest is only present for a few oscillations brief periods. This is a limitation of all tremor algorithms based on spectral estimation. Accurate estimate of a spectral peak typically requires 10 or more oscillations in each segment (2.5 s for a 4 Hz tremor). They are not well suited for measuring brief transients as occur with intention tremor and the finger-nose-finger task.

Our results demonstrate a moderate to strong correlation between TETRAS Performance and the inertial sensor data. However, we found that these correlations did not always reach significance. We believe this is due to the small sample size of the study.

It is also in part due to the way that the data were compared. We used the log inertial sensor values to compare to the TETRAS scores. However, the algorithm didn’t always detect tremor, in which case the inertial sensor value registered was zero. These values were excluded from the correlation analysis, which also decreased the sample size for particular comparisons (e.g. see kinetic condition on Fig. 7).

If the algorithm is unable to detect a spectral peak with a power fraction greater than the minimum threshold ($\rho _{min} = 0.1$), the algorithm does not estimate the tremor amplitude. If signal power over the expected tremor frequency range during that period, it indicates that no significant tremor is present. However, if there is profound other movement activity, the algorithm may not be able to detect tremor either because tremor is not present or because the tremor power is much smaller than the other activity. This approach helps reduce the rate of false positives, but possibly at the expense of false negatives during other vigorous activities.

One other limitation is that we limited our assessment to limb and truncal tremor, and excluded cranial measures of tremor (face, head, voice). However, we believe that this was necessary for feasibility reasons due to lack of ADL-ready wearable systems for cranial tremor quantification.

These results support the use of inertial sensors to objectively quantify tremor severity during a standardized exam following the TETRAS Performance scale, as they are easy to use and also help to eliminate subjectivity and bias that may occur and impact inter-rater and/or intra-rater reliability. This consistency in measurement would ensure accurate, precise recordings over time to track any subtle changes in tremor during patient follow-ups irrespective of timing between visits. Therefore, and given the range of tasks covered in the TETRAS assessment, we hypothesize that the demonstrated accurate and objective quantification of tremor may well translate to self-initiated movement outside a formal clinician-instructed exam. In addition, their low cost and ease of use would allow them to be available and used in many clinical sites.

The low cost and ability to send data using electronic means would also enable at-home tremor assessments. In circumstances where patients are required to stay home, have difficulty leaving the house, or live far from an availability movement disorder clinic, the ability to perform valid at-home data collection of prescribed movements can be an important way to monitor changes in tremor severity over time.

Under current practice, tremor quantification using TETRAS is performed in-office under the direction and supervision of a trained professional during a visit. This provides only a brief snapshot of the patient’s tremor, and perhaps may not capture key features of the tremor. For example, it’s possible that particular postures involved in the TETRAS assessment may not reflect the tremor as it is experienced at home in the context of longer recordings during a variety of activities of daily living. Similarly, variability in tremor frequency or amplitude may also occur throughout the day even for the same posture(s), and this may not be captured well within the short review period used for TETRAS.

We anticipate that the process for at-home recording will be relatively straightforward for patients, given that the devices can be easily attached to the wrist like a watch. Devices would be worn during waking hours, or whenever the patient felt comfortable doing so. The data would be collected and stored on the device, and subsequently analyzed using the algorithm upon return of the device. This would maximize the ability of the algorithm’s first step to estimate the tremor range for each individual.

The process by which this new algorithm first estimates the tremor range of the individual and then uses this to then more accurately detect tremor will help to ensure that readings are focused on the tremor and able to ignore other artifacts or noise that may be introduced to the signals while performing normal tasks of daily living. This can also be used to understand differences in tremor frequency during different tasks, as well as differences across limbs.

## V. Conclusion

We described a novel algorithm for quantifying tremor severity with inertial sensors for people with ET. This algorithm has several key advantages over previous algorithms. It uses a two-stage approach that estimates the average tremor frequency of the subject in the first stage and only detects tremor near that range in the second stage. This eliminates false positives that can occur when the tremor is detected over the full range of tremor frequencies that are possible for a subject population. It estimates the tremor amplitude as only the portion of signal power that is above the baseline. This provides for accurate tremor estimation even in the presence of other activity, as is common with action tremor that occurs in people with ET. The algorithm also estimates the tremor amplitude in physical units of translation (cm) and rotation (°)

We assessed the performance of the algorithm with both a robot arm in which the tremor amplitude was precisely controlled and with a clinical validation in which we compared the tremor amplitude to TETRAS, the most common clinical rating scale for ET. The findings we report here demonstrate that wearable inertial sensors with an appropriate algorithm can quantify tremor accurately even in the presence of other activities, and provides a strong step forward for at-home monitoring.
