# Errata to “Quantifying Tremor in Essential Tremor Using Inertial Sensors Validation of an Algorithm”

**DOI:** 10.1109/JTEHM.2020.3036539

**Published:** 2020-12-21

**Authors:** Patrick Mcgurrin, James Mcnames, Tianxia Wu, Mark Hallett, Dietrich Haubenberger

**Affiliations:** 1 National Institute for Neurological Disorders and Stroke, National Institutes of HealthBethesdaMD20892USA; 2 Department of Electrical and Computer EngineeringPortland State University 6685 PortlandOR97201USA; 3 Office of the Clinical DirectorNational Institute for Neurological Disorders and Stroke, National Institutes of Health,BethesdaMD20892USA

## Abstract

In the above article [1], the following disclosures should have appeared. Dietrich Haubenberger is now employed by Neurocrine Biosciences, Inc., San Diego, CA 92130, USA. James McNames is an employee of APDM, Portland, OR 97201, USA, a company that may have a commercial interest in the results of this research and technology. This potential conflict of interest has been reviewed and managed by OHSU.

In the above article [1], the following disclosures should have appeared. Dietrich Haubenberger is now employed by Neurocrine Biosciences, Inc., San Diego, CA 92130, USA. James McNames is an employee of APDM, Portland, OR 97201, USA, a company that may have a commercial interest in the results of this research and technology. This potential conflict of interest has been reviewed and managed by OHSU.

## References

[1] McGurrin P., Mcnames J., Wu T., Hallett M., and Haubenberger D., “Quantifying tremor in essential tremor using inertial sensors—Validation of an algorithm,” IEEE J. Transl. Eng. Health Med., vol. 9, 2020, Art. no. 2700110. 10.1109/JTEHM.2020.3032924 PMC7608862 33150096

